# Serum free light chain measurement aids the diagnosis of myeloma in patients with severe renal failure

**DOI:** 10.1186/1471-2369-9-11

**Published:** 2008-09-22

**Authors:** Colin A Hutchison, Tim Plant, Mark Drayson, Paul Cockwell, Melpomeni Kountouri, Kolitha Basnayake, Stephen Harding, Arthur R Bradwell, Graham Mead

**Affiliations:** 1 Department of Nephrology, University Hospital Birmingham, UK; 2 Department of Medical Sciences, University of Birmingham, UK; 3 Department of Immunology, University of Birmingham, UK; 4 IDRL, The Binding Site, Birmingham, West Midlands, UK

## Abstract

**Background:**

Monoclonal free light chains (FLCs) frequently cause rapidly progressive renal failure in patients with multiple myeloma. Immunoassays which provide quantitative measurement of FLCs in serum, have now been adopted into screening algorithms for multiple myeloma and other lymphoproliferative disorders. The assays indicate monoclonal FLC production by the presence of an abnormal κ to λ FLC ratio (reference range 0.26–1.65). Previous work, however, has demonstrated that in patients with renal failure the FLC ratio can be increased above normal with no other evidence of monoclonal proteins suggesting that in this population the range should be extended (reference range 0.37–3.1). This study evaluated the diagnostic sensitivity and specificity of the immunoassays in patients with severe renal failure.

**Methods:**

Sera from 142 patients with new dialysis-dependent renal failure were assessed by serum protein electrophoresis (SPE), FLC immunoassays and immunofixation electrophoresis. The sensitivity and specificity of the FLC ratio's published reference range was compared with the modified renal reference range for identifying patients with multiple myeloma; by receiver operating characteristic curve analysis.

**Results:**

Forty one patients had a clinical diagnosis of multiple myeloma; all of these patients had abnormal serum FLC ratios. The modified FLC ratio range increased the specificity of the assays (from 93% to 99%), with no loss of sensitivity. Monoclonal FLCs were identified in the urine from 23 of 24 patients assessed.

**Conclusion:**

Measurement of serum FLC concentrations and calculation of the serum κ/λ ratio is a convenient, sensitive and specific method for identifying monoclonal FLC production in patients with multiple myeloma and acute renal failure. Rapid diagnosis in these patients will allow early initiation of disease specific treatment, such as chemotherapy plus or minus therapies for direct removal of FLCs.

## Background

Immunoglobulin free light chains (FLCs) are by-products of immunoglobulin synthesis and in normal subjects are released into the circulation in small quantities [1]. The FLCs are then rapidly removed by renal clearance [2]. In patients with multiple myeloma, however, the clonal proliferation of plasma cells can produce FLCs in quantities thousands of times higher than normal [3]. These monoclonal FLCs often result in renal pathologies, most importantly cast nephropathy [4-6]. Indeed, multiple myeloma is the haematological malignancy most commonly associated with acute kidney injury (AKI) [7]. It has been proposed that the combination of multiple myeloma and AKI should be treated as a medical emergency with prompt diagnosis and intervention to avoid irreversible renal failure [8]. However, the standard screening tests for myeloma, serum protein electrophoresis (SPE) and urine Bence Jones protein analysis are not always requested or reported promptly.

Recently, immunoassays which measure the concentration of FLCs in serum have been incorporated into haematological screening algorithms for myeloma [9-11]. These FLC assays are automated and allow same-day analysis and reporting of results. With these assays, the presence of monoclonal FLC production is indicated when the ratio of kappa (κ) to lambda (λ) serum FLCs is outside the reference range of 0.26–1.65 [12]. The presence of an abnormal FLC ratio, suggestive of monoclonal FLCs production can occur in the settings of both intact immunoglobulin multiple myeloma and light chain only multiple myeloma. The identification of monoclonal protein production is not proof of multiple myeloma, but indicates that further investigations are required (principally a bone marrow biopsy and skeletal survey).

For patients presenting with AKI, more rapid identification of multiple myeloma may lead to earlier interventions and improved patient outcome. However, there are no reported evaluations of the diagnostic utility of FLC assays in this setting. One complicating factor is that patients with renal impairment can have κ/λ FLC ratios slightly above the reference range with no other evidence of monoclonal proteins [13,14]. This reflects a change in the dynamics of serum FLC clearance in renal failure. In normal subjects, the clearance of FLC from the serum is dominated by renal removal of FLCs which is preferential to the smaller, monomeric, κ molecules. This gives a shorter serum half-life for κ and a median κ/λ FLC ratio of approximately 0.6. As the kidneys fail, however, the non-preferential reticulo-endothial route forms an increasing proportion of the FLC clearance [15]. This results in a more similar serum half-life for the two FLCs and the FLC ratio therefore becomes increasingly influenced by the underlying production rates, by the plasma cells. There are approximately twice as many κ producing cells as there are λ cells [16] and this results in a ratio of total κ to total λ in the serum of approximately 1.8 [12].

As expected, FLC analysis of sera from 688 patients with pre-dialysis, chronic kidney disease but no evidence of monoclonal immunoglobulin production (by serum immunofixation electrophoresis) demonstrated the serum κ and λ FLC concentrations increased with decreasing renal function, FLC ranges: 3–251 mg/L and 1–251 mg/L, respectively. The κ/λ FLC ratio increased with each increasing chronic kidney disease stage, through stages: 1–5 (population's serum creatinine: 56–875 μmol/L; estimated GFR: 6–128 mL/min/1.73 m^2^). The median κ/λ FLC ratio of the population was 1.1 with a 100% range of 0.37–3.1 [17]. This change in the ratio could reduce the diagnostic utility of FLC analysis in renal impairment. We propose that modifying the κ/λ reference range to 0.37–3.1 may improve the diagnostic specificity when investigating patients with renal failure.

The aim of this study was to evaluate serum FLC measurement as a diagnostic tool for detecting monoclonal FLCs and underlying multiple myeloma, in patients with dialysis-dependent AKI. The sensitivity and specificity of the published reference range was compared with the proposed renal failure reference range.

## Subject and methods

### Patients

This study was undertaken in the departments of clinical immunology and the renal unit, at the University Hospital Birmingham as part of routine service development (audit reference: CA4-02015-08) and fully complies with the Declaration of Helsinki. All data analysis was coded and anonymised. Sera from patients who presented with new dialysis-dependent renal failure, to the renal unit at the University Hospital Birmingham, were screened for inclusion in a trial assessing the management of severe renal failure in multiple myeloma (COREC 05/Q2706/107, South Birmingham Research Ethics Committee), preliminary results of which have been reported previously [18].

Initiation of dialysis was by the consulting nephrologist for the following indications: uremia, hyperkalaemia, metabolic acidosis and fluid overload in the presence of renal failure, defined as an estimated glomerular filtration rate of less than 15 mL/min/1.73 m^2^; calculated using the MDRD equation [19]. Identification of the renal pathology was determined by the nephrologist following standard diagnostic pathways. Attribution of the cause of renal failure to multiple myeloma was based on a renal histology or in cases where a renal biopsy was contraindicated when all other potential causes were excluded. The clinical diagnosis of multiple myeloma was made by a consultant haematologist in accordance with international diagnostic criteria [20].

### Laboratory analysis

Serum samples were stored at -20°C until thawed for the current study; previous work has demonstrated the stability of FLC concentrations in urine samples over many years [21] and apparent stability in serum samples [12]. Serum protein electrophoresis (SPE) and immunofixation electrophoresis (IFE) were undertaken using the Sebia Hydragel 15/30 Protein kit and the Hydragel 4 Immunofixation PE kit on the Hydrasys system (Sebia, Lisses, France). Serum κ and λ FLC concentrations were measured by nephelometry, on a Dade-Behring BN™ II Analyser, using particle-enhanced, high-specificity, homogeneous immunoassays (Freelite™, The Binding Site, Birmingham, UK) [22]. FLC results were compared with the published reference range for the FLC ratio (κ/λ: 0.26–1.65) [12] and the proposed renal failure reference range (0.37–3.1). All sera were assessed with SPE and FLC immunoassays; samples with abnormal results were investigated further by IFE. Urine of patients with suspected multiple myeloma was assessed for monoclonal FLCs by immunofixation.

### Statistical analysis

Data were analysed using SPSS 14.0 for Windows and the Mann-Whitney *U *test was used to compare results from different patient groups. Receiver operating characteristic (ROC) curve analysis was used to examine the sensitivity and specificity of utilizing the standard reference range for the FLC ratio versus the proposed reference range.

## Results

The demographics and selected assay results for the patients are presented in table 1. There were no significant differences in the demographics of the AKI patients with and without myeloma. The patients with multiple myeloma however, had higher median serum creatinines at presentation.

**Table 1:** Dialysis populations: demographics and haematological data.

	Whole population (n-142)	Multiple myeloma patients (n-41)
Male – percentages	39%	56%
Ethnicity – percentages		
Caucasian	85%	85%
Afro-Caribbean	4%	5%
South-Asian	8%	10%
Other	3%	0%
Median age in years (range)	70 (19–88)	67 (38–84)
Median serum creatinine μmol/L (range)	529 (274–1758)	622 (302–3000)*
Myeloma type – percentages		
IgGκ		24%
IgGλ		22%
IgAκ		7%
IgAλ		15%
IgMλ		2%**
Free κ only		10%
Free λ only		19%
κ light chain class (overall) %		41%

*Serum creatinine significantly higher in multiple myeloma patients compared with the dialysis population as a whole, P < 0.02. **One patient with known chronic lymphocytic leukaemia, in remission, presented with acute renal failure and biopsy proven cast nephropathy. Investigation identified an intact IgM band on immunofixation electrophoresis with an associated λ FLC, bone marrow examination provided a haematological diagnosis of IgM myeloma.

**Figure 1:**
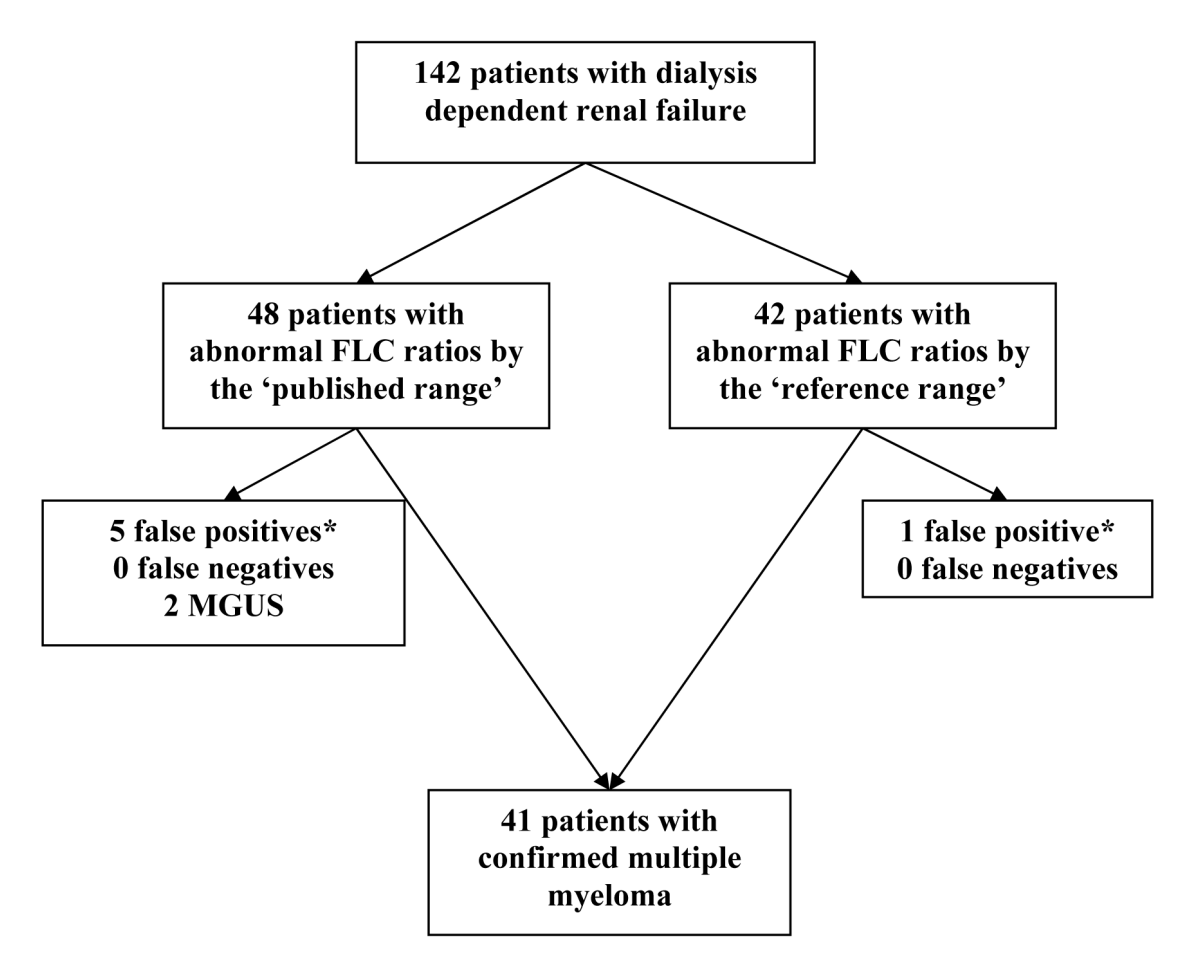
**Flow diagram of FLC results and myeloma diagnoses**. The proposed reference range reduced the number of false positive results. *false positive defined as patients with abnormal ratio and normal serum immunofixation. MGUS – monoclonal gammopathy of undetermined significance.

**Figure 2:**
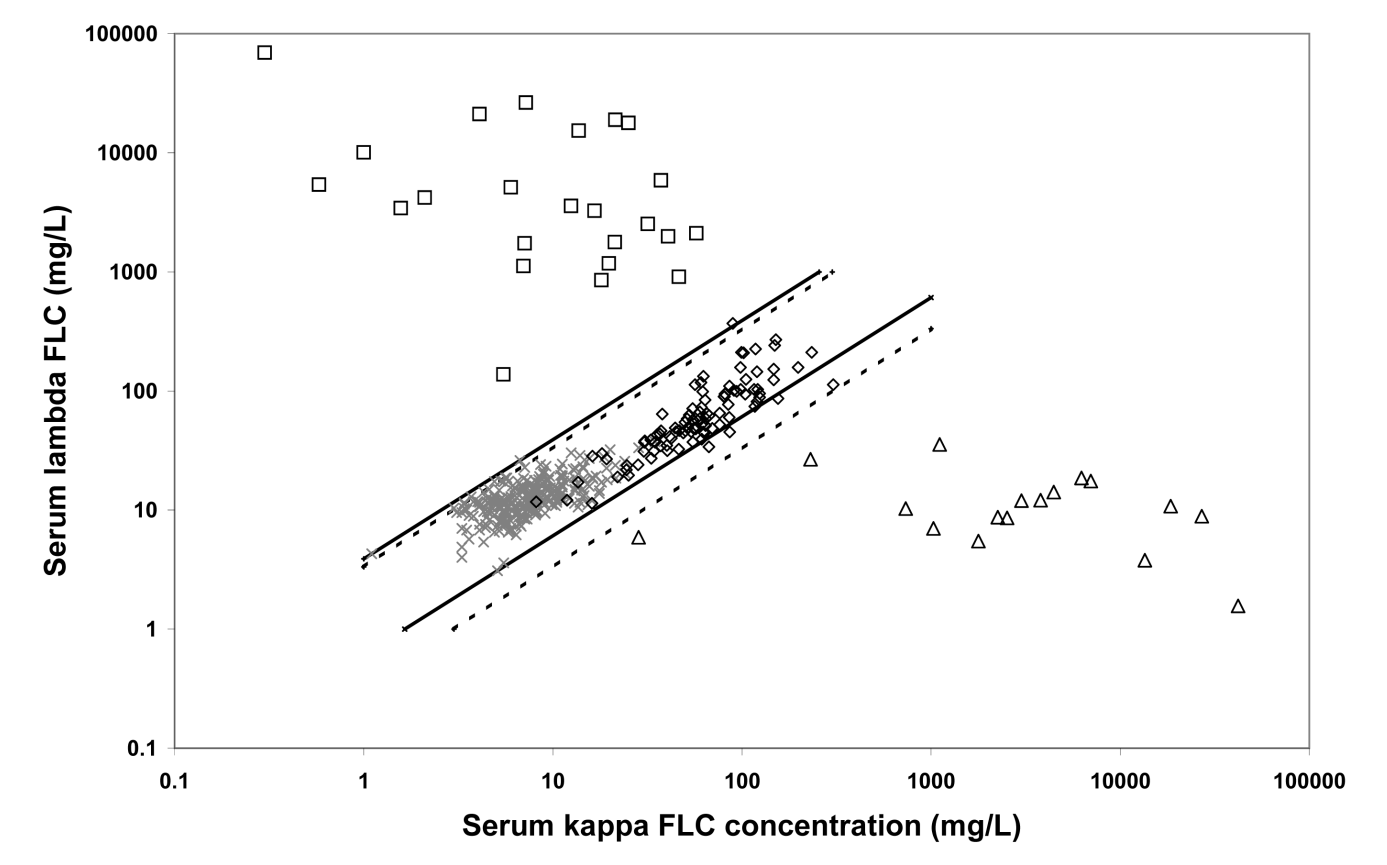
**Serum FLC concentrations in patients with dialysis-dependent, acute renal failure**. The FLC ratio easily distinguishes dialysis patients with κ (triangles) and λ (squares) multiple myeloma from patients with no evidence of monoclonal gammopathies (diamonds). Published normal control patients (crosses)^12 ^have significantly lower concentrations of polyclonal FLCs compared with the dialysis population (both P < 0.001).

**Figure 3:**
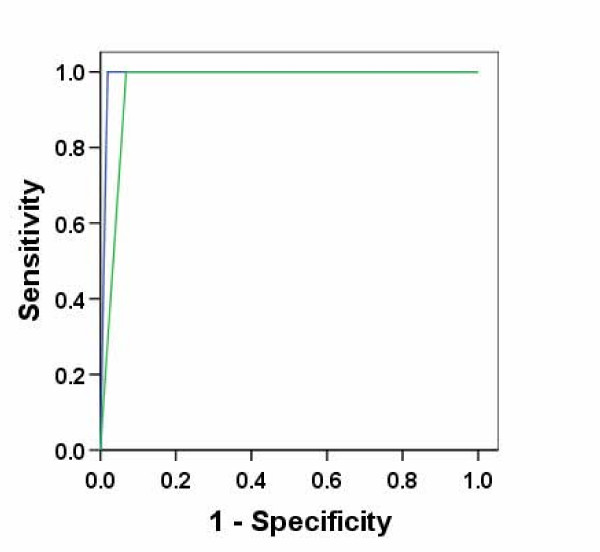
**Receiver operating characteristic curve (ROC) analysis of serum free light chain ratio analysis in patients with dialysis-dependent renal failure**. When using an abnormal serum FLC ratio as an indication of underlying multiple myeloma, comparison with the proposed renal reference range for the FLC ratio increased the area under the curve from that of 0.96 using the published reference range (green line, CI: 0.93–0.99; P < 0.001) to 0.99 with the new range (blue line, CI: 0.98–1.00; P < 0.001). Use of this new reference range did not change the sensitivity.

Forty-one of the 142 AKI patients had a clinical diagnosis of multiple myeloma (Figures 1 and 2). All of these 41 patients had abnormal FLC ratios by both the published reference range and the proposed reference range. The proposed reference range increased the specificity of the assay for diagnosis of multiple myeloma (99% versus 93%), with no loss in sensitivity (100%) and thus increased the area under the ROC curve (0.96 to 0.99; Figure 3).

Using the published reference range, seven patients had abnormal FLC ratios but no clinical diagnosis of multiple myeloma. Of these, two had monoclonal immunoglobulins identified by IFE and five did not; these five had ratios just outside the reference range, four above (1.79, 1.90, 1.97, 2.69) and one below (0.24). These ratios were markedly different from those of the patients with multiple myeloma in this population, with median ratios of 313 (range 4.8–26751) and 0.003 (0.05–0.000004), for κ and λ producing clones respectively. The two patients with abnormal FLC ratios and monoclonal immunoglobulin detected by IFE, but without a diagnosis of myeloma, had FLC ratios of 1.71 and 1.78. These two patients were classified as having monoclonal gammopathies of undetermined significance (MGUS); their renal diagnoses were acute tubular necrosis and renovascular disease, respectively. One further patient, with bilateral hydronephrosis, had an intact immunoglobulin MGUS detected by SPE and IFE but normal serum FLCs (ratio 0.95). When the proposed renal reference range for the FLC ratio was used, there was only one patient with an abnormal ratio (0.24) who did not have a diagnosis of myeloma.

Seventeen of the 41 multiple myeloma patients did not have adequate urine samples sent to the laboratory for analysis. Of the 24 who did, 10 had monoclonal κ FLCs detected and 13 had monoclonal λ. The remaining patient did not have monoclonal FLCs detected by urinary immunofixation, despite 1780 mg/L of κ FLCs measured in the serum and a renal diagnosis of cast nephropathy.

Renal biopsies were performed in 27 of the 41 patients with multiple myeloma. Twenty-five of the 27 had cast nephropathy as the principal diagnosis; of the two other biopsies, one showed acute interstitial nephritis the other acute tubular necrosis (ATN). Of the 13 myeloma patients who did not have a renal biopsy, one had bilateral hydronephrosis while the other 12 patients did not have biopsies undertaken because of clinical contraindications, predominately thrombocytopenia. The 25 myeloma patients with proven cast nephropathy had higher median serum concentrations of monoclonal FLCs than the four patients with other definitive renal diagnoses (the two with other histological diagnoses, the patient with bilateral hydronephrosis and another with severe sepsis and a clinical diagnosis of ATN). Median FLC ratios were: 3081 (range 31–26751) and 0.006 (range 0.0000–0.027) versus 6.7 (range 4.8–8.6) and 0.02 (0.0004–0.05), respectively. Median monoclonal FLC concentrations were: 10,250 mg/L (range 1,030–69,430) versus 1,068 mg/L (range 28–3,440), respectively. These differences did not reach significance (P = 0.13, 0.5 and 0.09, respectively).

The renal diagnoses patients with AKI, who were not diagnosed with myeloma were: non-diabetic glomerular nephritis (17.5%), diabetic kidney disease (15.5%), acute tubular necrosis (9.5%), renal vascular disease (15%), interstitial nephritis (5%), urinary tract pathologies (15.5%) and other (17.5%). The median serum polyclonal FLC concentrations for these patients were: κ-60.7 mg/L (range 8.2–304); λ-56.4 mg/L (range 11.4–370) and the median serum FLC ratio was 1.0 (range 0.24–2.69).

## Discussion

The purpose of this study was to assess serum free light chain immunoassays as an aid in the diagnosis of multiple myeloma in patients with severe renal failure. All 41 patients who were diagnosed with multiple myeloma were identified as abnormal using the assays. The assays indicate the presence of monoclonal FLCs by comparing the quantitative measurement of κ FLCs with λ FLCs, as a κ/λ FLC ratio. In patients with monoclonal κ FLC production the ratio is increased and in patients with monoclonal λ FLC the ratio is decreased. The sensitivity of the assays in this setting (100%) was not unexpected as previous studies have reported greater sensitivity for serum versus urine detection of monoclonal FLC in both myeloma [3,23] and AL amyloidosis [24,25].

Before serum FLC assays became available, urine analysis was the preferred method for identifying monoclonal FLC production in routine haematological screens for multiple myeloma and other lymphoproliferative disorders. However, the collection of urine samples, particularly 24 hour collections, is frequently problematic. In one screening study, urine samples were lacking for more than half the population [13] and in our study population urine samples were only provided for 24 of 41 myeloma patients. In an analysis of 428 patients with monoclonal FLCs in their urine, Katzmann *et al *[25] found that the combination of serum electrophoresis tests and serum FLC analysis identified all patients requiring treatment and could, therefore, remove the requirement for urine analysis when screening [25]. Notably, the urine from one of the myeloma patients in our study was reported as normal despite a clearly abnormal serum FLC concentration. This study, therefore, provides further evidence that serum FLC assays may have greater utility for identifying monoclonal FLC production than urine analysis.

The utility of a screening assay in practice however, is a function of its specificity as well as its sensitivity. In patients with renal failure, as glomerular filtration reduces, renal clearance of all FLCs will decrease. This results in longer serum half-lives and an increase in the κ/λ FLC ratio. Previous work we have undertaken demonstrated that in patients with renal failure, with no evidence of monoclonal proteins, the median FLC ratio was increased to 1.1 (range 0.37–3.1) from that of the published control population of 0.58 (0.26–1.65) [17]. We hypothesized that extending the reference range for the FLC ratio, to take into account this influence of renal function, would increase the specificity of the assay in patients with dialysis-dependent renal failure. Use of the published reference range for the FLC ratio, 0.26–1.65, gave the assay a specificity of 93% for patients with myeloma. This improved to 99% with the proposed extended reference range (0.37–3.1), indicating the new range may have a practical benefit by reducing the number of false positives.

Interpretation of FLC ratios between 1.65 and 3.0 is currently difficult. We would propose checking the patient's renal function. If normal, a ratio in this range may be indicative of a monoclonal process and further laboratory and clinical investigation will be appropriate. If the renal function is abnormal, a ratio in the range of 1.65–3.0 is probably a consequence of the renal impairment; however further investigation of some patients may be appropriate, particularly if AL-amyloidosis is suspected.

An interesting observation of this study was that the patients with cast nephropathy had higher absolute levels of the monoclonal free light chain type than the myeloma patients with other renal pathologies. Although this difference did not reach significance it adds further evidence to the findings of Bergner *et al *who demonstrated that urinary FLC concentrations are higher in patients with cast nephropathy compared with other FLC related renal pathologies [26].

As with the findings of previous studies, cast nephropathy was the predominant cause of dialysis-dependent renal failure in the patients with multiple myeloma who had biopsies reported (25/27) [4-6]. Historically, myeloma patients with biopsy proven cast nephropathy have less than 25% chance of renal recovery [5,27-29] and a significantly worse overall survival [30]. Early reversal of the renal failure however, improves patient survival [30,31]. Novel therapies, currently under evaluation, aim to increase these renal recovery rates and patient survival. The focus of the new treatments is to rapidly reduce serum FLC concentrations, by either effective chemotherapy alone [32,33] or in combination with direct removal of FLCs by high-cut-off haemodialysis [31]. Success however, is likely to depend upon early diagnosis and intervention; as animal models have indicated that within one month of obstruction, by a cast, irreversible damage has occurred to the nephron [34]. The role of serum FLCs in management of patients with multiple myeloma and renal failure may expand beyond that of a diagnostic tool and management guide to that of an independent indicator of prognosis as eloquently demonstrated by Kyrtsonis *et al *in the general myeloma population [35].

## Conclusion

The measurement of serum FLCs can be a practical and highly sensitive aid in the identification of myeloma as the underlying pathology in patients with severe renal failure. Using an extended renal failure reference range for the FLC ratio increased the specificity of the assays. The diagnostic accuracy of these assays and their rapid laboratory turn-around time should aid nephrologists in their assessment of acute renal failure.

## Competing interests

This study was designed and undertaken by the physicians based in the renal unit at the University Hospital Birmingham in collaboration with the department of clinical immunology at the University of Birmingham. Technical support and the provision of FLC immunoassays were provided by The Binding Site Ltd, Birmingham, UK. Dr Harding is an employee and Dr Mead and Professor Bradwell are directors of The Binding Site.

## Authors' contributions

CAH designed the study, undertook the data analysis and wrote the manuscript. TP and MD contribute to the design of the study, identification of patients and reviewed the manuscript. PC was involved with the design of the study, data analysis and writing of the manuscript. MK and KB assisted with data collection. SH carried out the immunoassays. ARB reviewed the study design and the manuscript. GM reviewed the study design and was fully involved with the data analysis and writing the manuscript. All authors read and approved the final manuscript.

## Pre-publication history

The pre-publication history for this paper can be accessed here:


